# The Genome of the Predominant Equine *Lactobacillus* Species, *Lactobacillus equi*, Is Reflective of Its Lifestyle Adaptations to an Herbivorous Host

**DOI:** 10.1128/genomeA.01155-13

**Published:** 2014-01-16

**Authors:** Michelle M. O’Donnell, Hugh M. B. Harris, Paul W. O’Toole, R. Paul Ross

**Affiliations:** Teagasc Food Research Centre, Moorepark, Fermoy, County Cork, Irelanda; Department of Microbiology, University College Cork, Cork, County Cork, Irelandb

## Abstract

We report the draft genome sequence of *Lactobacillus equi* strain DPC6820, isolated from equine feces. *L. equi* is a predominant *Lactobacillus* species in the horse hindgut microbiota. An examination of the genome identified genes and enzymes highlighting *L. equi* adaptations to the herbivorous gastrointestinal tract of the horse, including fructan hydrolases. This genome sequence may help us further understand the microbial ecology of the equine hindgut and the influence lactobacilli have on it.

## GENOME ANNOUNCEMENT

*Lactobacillus equi* is a lactic acid bacterium found in the gastrointestinal tracts of horses (1) and which, along with *Lactobacillus hayakitensis* and *Lactobacillus equigenerosi*, has been identified as the predominant lactobacillus of the equine hindgut (2). *L. equi* was isolated from a fecal sample of a healthy Irish Thoroughbred racehorse. The sequence data were obtained using the Illumina HiSeq 2000 reversible dye terminator system (Macrogen, Seoul, South Korea), with read lengths of 101 bp. The HiSeq system paired-end sequencing strategy generated 36,133,338 reads (3,649,467,138 bp). Two hundred fifty-four scaffolds containing 273 contigs were assembled, corresponding to 34,664,201 reads from the HiSeq system (3,501,084,301 bp), which represents 1,608-fold genome coverage based on an estimated genome size of 2.19 Mb. The N_50_ score for the assembly estimating contig length was 39,802 bp. The draft *L. equi* genome includes 2,187,681 bases (G+C content of 39.16%). It comprises 2,263 predicted genes or coding sequences (CDS). Eight rRNA operons and 68 predicted tRNAs, representing all 20 amino acids, were identified in the genome. Functions were predicted for 76% of the *L. equi* chromosomal genes. The functional assignment of the predicted genes was completed using Metagene (3) to predict open reading frames (ORFs) and BLASTp to annotate them using the NCBI database (4).

### Nucleotide sequence accession numbers.

This whole-genome shotgun project has been deposited at DDBJ/EMBL/GenBank under the accession no. AWWH00000000. The version described in this paper is version AWWH01000000.
